# Whole-genome epidemiology, characterisation, and phylogenetic reconstruction of *Staphylococcus aureus* strains in a paediatric hospital

**DOI:** 10.1186/s13073-018-0593-7

**Published:** 2018-11-13

**Authors:** Serena Manara, Edoardo Pasolli, Daniela Dolce, Novella Ravenni, Silvia Campana, Federica Armanini, Francesco Asnicar, Alessio Mengoni, Luisa Galli, Carlotta Montagnani, Elisabetta Venturini, Omar Rota-Stabelli, Guido Grandi, Giovanni Taccetti, Nicola Segata

**Affiliations:** 1 Centre for Integrative Biology, University of Trento, Trento, Italy; 2 Cystic Fibrosis Center, Interdisciplinary Specialist Department, Anna Meyer Children’s University Hospital, Florence, Italy; 3 Department of Biology, University of Florence, Florence, Italy; 4 Department of Health Sciences, University of Florence, Florence, Italy; 5 Infectious Diseases Unit, Anna Meyer Children’s University Hospital, Florence, Italy; 6 Department of Sustainable Agro-Ecosystems and Bioresources, Fondazione Edmund Mach, San Michele all’Adige, Italy

**Keywords:** *Staphylococcus aureus*, Microbial genomics, Microbial epidemiology, Bacterial pathogens

## Abstract

**Background:**

*Staphylococcus aureus* is an opportunistic pathogen and a leading cause of nosocomial infections. It can acquire resistance to all the antibiotics that entered the clinics to date, and the World Health Organization defined it as a high-priority pathogen for research and development of new antibiotics. A deeper understanding of the genetic variability of *S. aureus* in clinical settings would lead to a better comprehension of its pathogenic potential and improved strategies to contrast its virulence and resistance. However, the number of comprehensive studies addressing clinical cohorts of *S. aureus* infections by simultaneously looking at the epidemiology, phylogenetic reconstruction, genomic characterisation, and transmission pathways of infective clones is currently low, thus limiting global surveillance and epidemiological monitoring.

**Methods:**

We applied whole-genome shotgun sequencing (WGS) to 184 *S. aureus* isolates from 135 patients treated in different operative units of an Italian paediatric hospital over a timespan of 3 years, including both methicillin-resistant *S. aureus* (MRSA) and methicillin-sensitive *S. aureus* (MSSA) from different infection types. We typed known and unknown clones from their genomes by multilocus sequence typing (MLST), Staphylococcal Cassette Chromosome *mec* (SCC*mec*), Staphylococcal protein A gene (*spa*), and Panton-Valentine Leukocidin (PVL), and we inferred their whole-genome phylogeny. We explored the prevalence of virulence and antibiotic resistance genes in our cohort, and the conservation of genes encoding vaccine candidates. We also performed a timed phylogenetic investigation for a potential outbreak of a newly emerging nosocomial clone.

**Results:**

The phylogeny of the 135 single-patient *S. aureus* isolates showed a high level of diversity, including 80 different lineages, and co-presence of local, global, livestock-associated, and hypervirulent clones. Five of these clones do not have representative genomes in public databases. Variability in the epidemiology is mirrored by variability in the SCC*mec* cassettes, with some novel variants of the type IV cassette carrying extra antibiotic resistances. Virulence and resistance genes were unevenly distributed across different clones and infection types, with highly resistant and lowly virulent clones showing strong association with chronic diseases, and highly virulent strains only reported in acute infections. Antigens included in vaccine formulations undergoing clinical trials were conserved at different levels in our cohort, with only a few highly prevalent genes fully conserved, potentially explaining the difficulty of developing a vaccine against *S. aureus*. We also found a recently diverged ST1-SCC*mec*IV-*t127* PVL− clone suspected to be hospital-specific, but time-resolved integrative phylogenetic analysis refuted this hypothesis and suggested that this quickly emerging lineage was acquired independently by patients.

**Conclusions:**

Whole genome sequencing allowed us to study the epidemiology and genomic repertoire of *S. aureus* in a clinical setting and provided evidence of its often underestimated complexity. Some virulence factors and clones are specific of disease types, but the variability and dispensability of many antigens considered for vaccine development together with the quickly changing epidemiology of *S. aureus* makes it very challenging to develop full-coverage therapies and vaccines. Expanding WGS-based surveillance of *S. aureus* to many more hospitals would allow the identification of specific strains representing the main burden of infection and therefore reassessing the efforts for the discovery of new treatments and clinical practices.

**Electronic supplementary material:**

The online version of this article (10.1186/s13073-018-0593-7) contains supplementary material, which is available to authorized users.

## Background

*Staphylococcus aureus* is a bacterium commonly found on the skin (15%), in the nostrils (27%), and in the pharynx (10–20%) of healthy adults [1–3], but it is also the cause of a number of diseases, whose severity ranges from common community-associated skin infections to fatal bacteraemia [3–5]. *S. aureus* is a leading cause of surgical, device-related, and pleuropulmonary infections, which can result into life-threatening infective endocarditis or even sepsis [6]. The mortality of *S. aureus* invasive infections was extremely high (> 80%) in the pre-antibiotic era [6, 7], and only the introduction of penicillin at the beginning of the 1940s was able to contain it. However, resistant strains carrying a penicillinase/beta-lactamase quickly emerged [8–10], and more than 90% of current human-associated isolates are resistant to penicillin [6]. Similarly, the introduction of the penicillinase-resistant antibiotic methicillin was quickly followed by the emergence of methicillin-resistant *S. aureus* (MRSA) clones [11–13]. *S. aureus* is capable of acquiring resistance to virtually every antibiotic that has entered clinical use [14, 15], including recently developed agents like daptomycin and linezolid [16, 17] and the last resort antibiotic vancomycin [18, 19]. In 2017, the World Health Organization has listed vancomycin-intermediate and vancomycin-resistant MRSA among the high priority pathogens for research and development of new antibiotics [20].

*S. aureus*’s ability to spread worldwide and to cause outbreaks in both hospitals and the community [21, 22] has fostered the study of its global epidemiology [3, 15, 23–25]. Some lineages are very prevalent worldwide (e.g. CC5 and CC8) [24], whereas others have a more localised spreading range, like the CC5 ST612 clone, which has been found only in South Africa and Australia [24, 26]. MRSA prevalence is also highly geographically variable, ranging from < 1% in some Northern European countries to > 50% in some American and Asian countries, with livestock-associated MRSA disseminating in the last two decades [24]. Newly emerging highly pathogenic and pandemic clones have also been globally characterised [27, 28] and are often the results of recombination events as in the case of the ST239-SCC*mec*III clone [25, 27, 29]. *S. aureus* investigations have however often underestimated the importance of non-MRSA clones, usually considering only hypervirulent or specifically relevant methicillin-sensitive *S. aureus* (MSSA) lineages [15], even though MSSA is the most common cause of surgical site infection [30, 31] and one of the major nosocomial pathogens [15].

Untargeted profiling of the entire *S. aureus* population in a given site or area is as important as its global epidemiology, and it is crucial for surveillance and prevention of local outbreaks. Some studies have for instance unbiasedly assessed the local epidemiology of nosocomial *S. aureus*, suggesting that this pathogen is only rarely transmitted from nurses to hospitalised patients in presence of adequate infection prevention measures [32] and that the community acts as major source of nosocomial MRSA [33]. Studies surveying the whole *S. aureus* population in hospitals have however focused on single aspects, like the diversity of the population, its virulence and resistance traits, and its transmission in presence of an outbreak [34–39] or in non-emergency conditions [40–42]. Despite the large body of researches on *S. aureus*, studies addressing a whole *S. aureus* infective population at a given site through whole genome sequencing to simultaneously look at the epidemiology, phylogenetic reconstruction, genomic characterisation, and transmission pathways of infective clones are currently limited [43]. Expanding these types of studies will be crucial for an in-depth global monitoring of *S. aureus*.

Here we report an in-depth epidemiological and genomic investigation of *S. aureus* infections in a paediatric hospital in Italy. With a whole-genome sequencing approach, we reconstructed the phylogenies of the clones in the cohort, characterised known clones and variants, screened for resistance and virulence genes, and tested for the presence of an outbreak. This allowed us to appreciate the high diversity of the *S. aureus* community, with 80 different lineages, variability of the resistance cassettes, and uneven conservation of various antigens previously clinically tested for vaccine development. We further report an increased prevalence of highly resistant and lowly virulent clones in chronic infections, and the rise of a newly emerging clone already reported in other hospitals. Overall, our results highlight the complexity of *S. aureus* epidemiology and advocate the need for wider genome-based analysis.

## Materials and methods

### Sample collection and *S. aureus* isolation

Samples were collected at Anna Meyer Children’s University Hospital (Florence, Italy) from 160 patients from January 2013 to December 2015. Metadata were also collected (Additional file 1: Table S1). We analysed samples obtained from the most common sites of infection for *S. aureus*, namely airways (bronchial aspirates, sputum or oropharyngeal and nasal swabs) or from soft-tissue and skin lesions. All samples were processed for the detection of bacteria using selective (Mannitol Salt Agar 2, bioMérieux) and chromogenic culture media for MRSA (BBL™ CHROMagar™ MRSA II, Becton Dickinson). In order to confirm species-level identification, mass spectrometry analysis was performed using matrix-assisted laser desorption/ionisation time of flight (MALDI-TOF) (VITEK® MS, bioMérieux). Antibiotic susceptibility was evaluated using the automated system VITEK®2 (bioMerieux) with the card AST-P632 (see Additional file 1: Table S1 for antibiograms). All identified strains were stored at − 80 °C for the following molecular analysis.

### Molecular characterisation of *S. aureus* and MRSA isolates

DNA extraction was performed from pure *S. aureus* cultures after 24 h of incubation at 37 °C on Columbia agar + 5% sheep blood (bioMérieux) using QIAamp DNA Mini Kit (cat. num. 51306, QIAGEN, Netherlands) according to the manufacturer’s specifications. DNA was purified using Agencourt AMPure XP (Beckman Coulter, California, USA) according to the manufacturer’s specifications. Extracted DNA was stored at − 20 °C for further analyses.

In order to determine the potential virulence of SA/MRSA strains, a specific PCR assay for the presence of the gene (*lukS-lukF*) encoding for the Panton-Valentine Leukocidin (PVL) was set up following a previously published protocol [44]. The *mecA* gene and other loci of the SCC*mec* cassette were analysed using different multiplex PCR. The protocol suggested by Milheirico et al. [45] has been used as a screening test for most frequent SCC*mec* cassettes types (types I, II, III, IV, V, and VI) and then confirmed with other methods in equivocal cases [45–48].

PCR-based multilocus sequence typing (MLST) was carried out with 25 μl reaction volumes containing 2 μl of chromosomal DNA, 20 μM of each primer, 1 U of Taq DNA polymerase (Super AB Taq, AB analitica), 2.5 μl of 10× PCR buffer (supplied with the Taq polymerase), 1.5 μM MgCl_2_, and 250 μM each deoxynucleoside triphosphates. The PCR was performed with an initial 5-min denaturation at 95 °C, followed by 30 cycles of annealing at 55 °C for 1 min, extension at 72 °C for 1 min, and denaturation at 95 °C for 1 min, followed by a final extension step of 72 °C for 5 min. The amplified products were purified and then amplified with the BigDye® Terminator v3.1 Cycle Sequencing Kits (Applied Biosystem) with the primers used in the initial PCR amplification. The sequences of both strands were determined with an ABI Prism 310 DNA sequencer. Isolates with the same ST have identical sequences at all seven MLST loci.

### Isolates sequencing and data pre-processing

DNA libraries were prepared with Nextera XT DNA Library Preparation Kit (Illumina, California, USA). Quality control was performed with Caliper LabChip GX (Perkin Elmer) prior to shotgun sequencing with MiSeq (Illumina, California, USA), with an expected sequencing depth of 260 Mb/library (expected coverage > 80×). One hundred twenty-nine million reads were generated (704 thousand reads/sample s.d. 349 thousand).

Sequences were pre-processed by removing low-quality (mean quality lower than 25) or low-complexity reads, reads mapping to human genome or to large and small ribosomal units of bacteria, fungi and human, and known contaminants (e.g. phiX174, Illumina spike-in). All genomes are available at the NCBI Sequence Read Archive (BioProject accession number PRJNA400143).

### Genome assembly and annotation

Pre-processed reads were de novo assembled using SPAdes version 3.6.1 [49] and discarding contigs shorter than 1000 nt. We selected for our analysis only reconstructed genomes with an N50 > 50,000. We obtained high-quality genomes (N50 > 50,000 and less than 150 contigs) for 135 of the 160 patients enrolled. Genomes belonging to the remaining 25 patients were excluded from further analyses. Genomes were annotated with Prokka version 1.11 [50] using default parameters and adding --addgenes and --usegenus options.

### Genome alignment/phylogenetic analysis

**Fig. 1:**
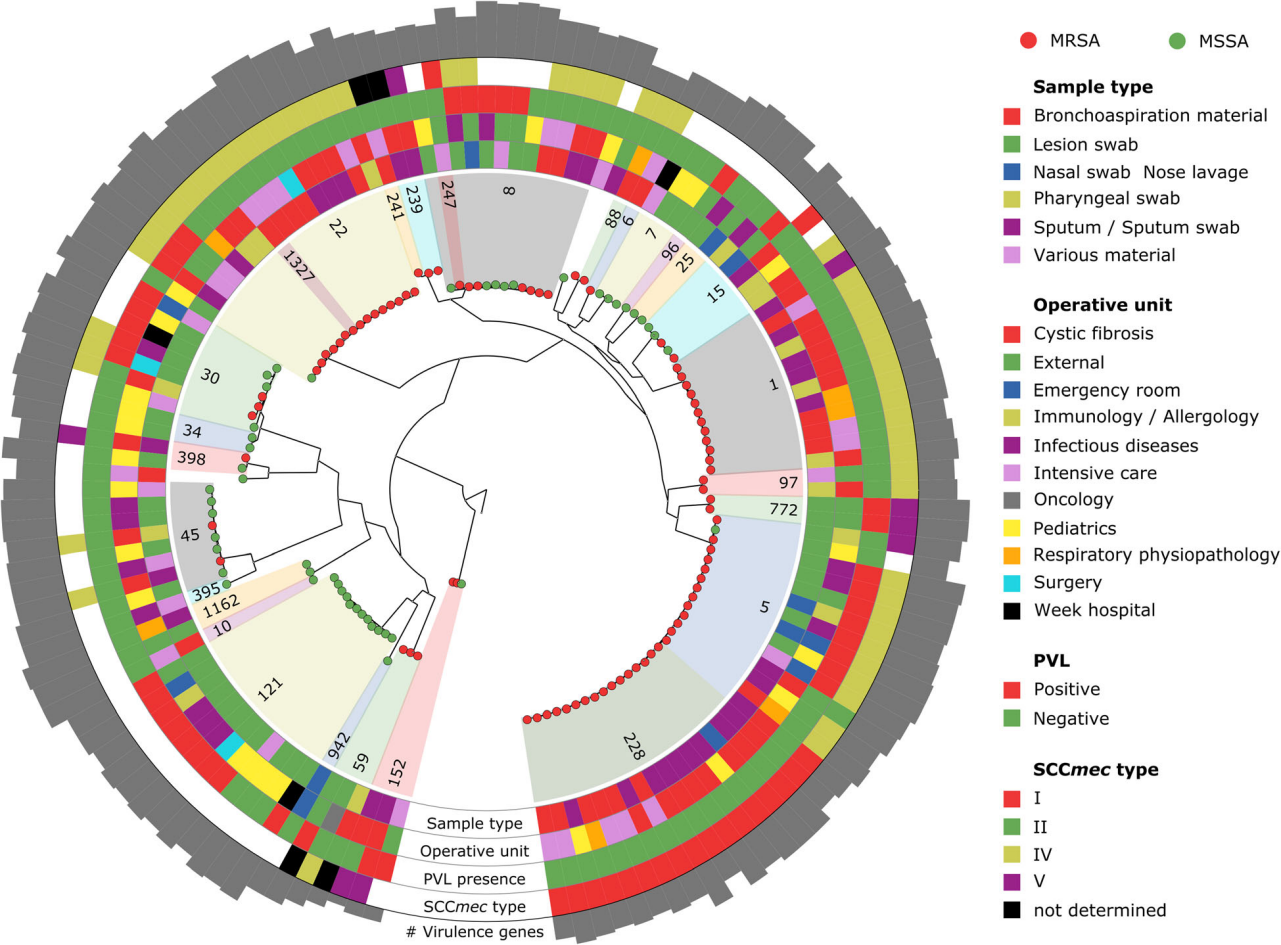
Phylogenetic tree of the whole cohort. Phylogenetic tree based on the 1464 core genes (1,194,183 bases) of the 135 single-patient *S. aureus* isolates. STs are distinguished by means of numbers and background colours in the inner ring. Sample type, operative unit, PVL presence, and SCC*mec* type are colour-coded in the following rings. On the outermost ring, the number of virulence genes is reported as bar plot (total considered = 79)

**Fig. 2:**
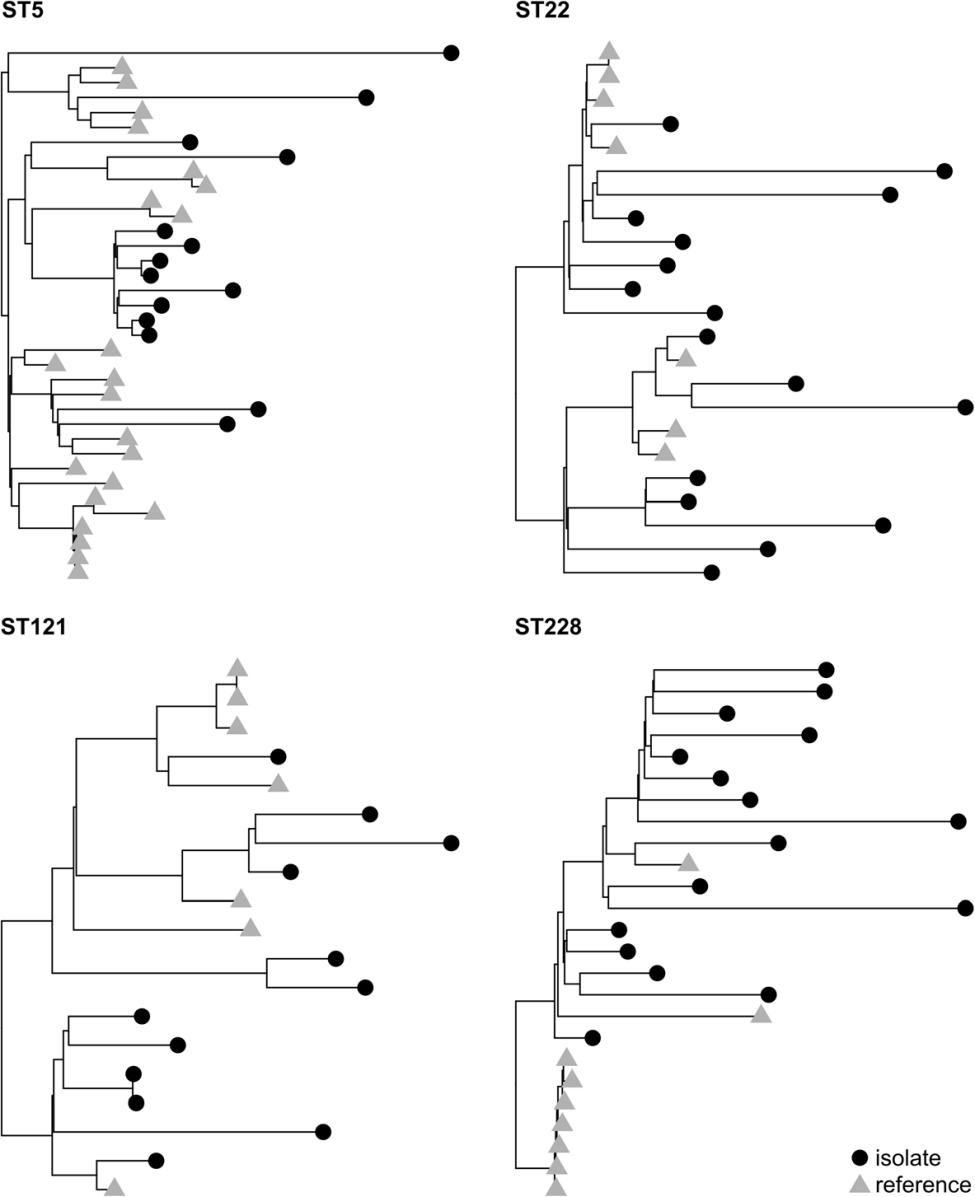
Whole-genome maximum likelihood phylogenetic trees of the four most relevant STs. All available reference genomes for ST22, ST121, and ST228 have been included. For ST5, 1478 reference genomes were available, but only 24 were included for the sake of clarity. The phylogenetic tree of ST1 and available reference genomes was also produced, but it is not reported here to avoid overlapping with Figure 5

The sets of 1464 concatenated genes used as input for constructing whole cohort (Fig. 1) and strain (Fig. 2) phylogenetic trees were generated using Roary version 3.4.2 [51]. Maximum likelihood trees were inferred with RAxML version 8.0.26 [52] using a GTR replacement model with four discrete categories of Gamma. Support at nodes was estimated using 100 bootstrap pseudo-replicates (option “-f a”). The phylogenetic tree in Additional file 2: Figure S1 was inferred using the presence-absence binary matrix of the core and accessory genes computed with Roary version 3.4.2 [51] in RAxML version 8.0.26 [52] with option “-m BINGAMMA”. Phylogenetic analyses were conducted using only one single isolate per patient; when multiple isolates from different timepoints of the same patient were available, the reconstructed genome with the highest N50 and the lowest number of contigs was selected. In most cases (*n* = 30), patients maintained the same ST over time; in discrepant cases (*n* = 2), we selected the most prevalent clone.

### In silico sequence type (ST), SCC*mec*, and *spa*-type identification

In order to assign SCC*mec* type also to equivocal cases and to confirm PCR-based SCC*mec* typing, the same set of primers [45] and other primer sets [53, 54] were mapped to reconstructed genomes by BLAST [55]. In most cases, the two methods were consistent. In discordant cases, PCR was repeated. Sequence typing and *spa*-typing were conducted using MetaMLST [56] and the DNAGear software [57] respectively.

Many isolates were not assigned a *spa*-type because of the limitations of short-read shotgun sequencing in repeated regions, which cause problems in genome assembly.

### Virulence factors and resistance gene analysis

Selected virulence factors and resistance genes (as in [58]) were searched for by mapping reference genes (Additional file 3: Table S2) to all reconstructed genomes with BLAST [55] with the following parameters [−evalue 1e−10 -perc_identity 90 -gapopen 5 -gapextend 5] with a match > 75%. Virulence genes to be searched for were selected on the basis of a careful literature review for their clinical relevance [59–84].

### Analysis of genes with available vaccine targets

Genes of interest were identified as those *S. aureus* vaccine candidates that had already entered clinical trials (according to http://clinicaltrials.gov as of January 2018), and those candidates that showed promising results in preclinical trials. For each genome, we extracted the reference sequences using BLAST [55] with default parameters. Extracted genes were pairwise globally aligned with the reference and evaluated for synonymous and non-synonymous single-nucleotide variation (SNVs), insertions, and/or deletions.

### Bayesian divergence estimates

We estimated divergence times of ST1 SCC*mec*IV t127 PVL− clones using BEAST2 [85] and the core genome (core genes = 1464). We defined the best fitting model priors by testing the combination of three clock models (uncorrelated relaxed exponential, uncorrelated relaxed lognormal, and strict), three demographic models (birth-death, coalescent Bayesian skyline, and constant), and two substitution models (HKY - Hasegawa, Kishino, Yano and generalised time reversible). Bayesian Markov chain Monte Carlo were run for 500 Mio. generations and sampled every 1000 generations. We chose the combination of models that resulted in the highest Bayes factor after parameter correction using AICM in Tracer (see Additional file 4: Table S3).

### Statistical tests

Associations between STs/virulence genes/antibiotic resistance markers and sample/operative unit types were found by performing Fisher’s exact test between the class of interest and the remaining set of samples.

## Results and discussion

We investigated the epidemiology and the whole-genome genetics of *S. aureus* isolated from multiple operative units of the same paediatric hospital in Italy (Meyer’s Children Hospital, Florence). Two hundred thirty-four *S. aureus* isolates from 160 patients were retrieved from diverse clinical specimens, tested for antibiotic susceptibility, and subjected to whole-genome sequencing (see Materials and methods). The study produced 184 high-quality reconstructed *S. aureus* genomes with a N50 larger than 50,000 and less than 250 contigs (Additional file 1: Table S1). Downstream analyses are focused on the 135 high-quality strains recovered from distinct patients.

### Genome sequencing highlights the presence of common clonal complexes and five newly sequenced clones

We first performed a whole-genome phylogenetic analysis to investigate the population structure of *S. aureus* in our cohort. The phylogeny was built using one isolate for each patient (*n* = 135) and using the 1464 core genes representing a core genome of > 1.19 M bases (see Materials and methods and Fig. 1). The genomic diversity of *S. aureus* is highlighted by the relatively large number of accessory genes even in a limited cohort of clinical isolates (*n* = 6909 from a pangenome of 8373 (Additional file 2: Figure S2), in concordance with a recent study based on the pangenome of 64 strains from different ecological niches [86]. The gene presence/absence phylogenetic model considering both core and genes confirmed the structure of the one built on the core genome alone, with however a slightly higher strain-diversity for isolates belonging to the same ST (Additional file 2: Figure S1). Despite this diversity, we found the presence of a reduced set of closely related strains in the cohort (Fig. 1) mostly associated with distinct multilocus sequence typing clones (STs) [87] (see Materials and methods). We identified a total of 29 different STs, with five of them—ST228, ST22, ST5, ST121, and ST1—found in at least 12 patients (Table 1 and Additional file 1: Table S1) with evidence of ST replacement in only one patient (Patient 091 switching from ST228 to ST22) of the 32 patients sampled at multiple timepoints. This longitudinal strain consistency was confirmed by whole-genome analysis (mean intra-patient variability = 56.42 SNVs), for which the replacing event in Patient 091 accounted for 6238 SNVs between the 2013 and 2016 isolates, 0.22% of the genome. The 29 identified STs belong to 14 clonal complexes (CCs), with the five most prevalent CCs (CC5, CC22, CC8, CC1, and CC121) comprising more than 60% of the isolates. *Spa*-typing [57] further refined the typing resolution: we found 44 distinct *spa*-types (Additional file 1: Table S1), with t001, t002, t008, and t127 being the most prevalent (i.e. present in > 4 isolates, Table 1). We also investigated the presence of the Panton-Valentine Leukocidin (PVL), a two-component prophage virulence factor allowing *S. aureus* to escape from the host immune system, that was found in 27.4% of the samples (Additional file 1: Table S1).

**Table 1:** Genomic characteristics of the different STs, including SCC*mec* and *spa*-type, presence of PVL, genome length, N50 (shortest sequence length at 50% of the genome), and number of contigs, coding DNA sequences (CDS), and genes

ST	CC	# isolates (MRSA)	Predominant SCC*mec* type (# isolates)	Predominant *spa*-type (# isolates)	# PVL+	Avg. genome length (bp)	Avg. # contigs	Avg. N50	Avg. # CDS	Avg. # genes
1	1	12 (12)	IV (11)	t127 (3)	0	2,814,074.3	29.4	326,193.0	2601.3	2666.8
772		2 (2)	V (2)	t657 (2)	2	2,768,135.0	46.0	208,282.5	2538.0	2605.0
5	5	14 (13)	IV (10)	t002 (5)	8	2,785,946.1	39.4	250,660.3	2580.1	2640.5
228		16 (16)	I (16)	t001 (5)	0	2,837,918.4	81.9	87,688.4	2639.8	2700.7
228	6	1 (1)	IV (1)	t5238 (1)	0	2,796,820.0	40.0	150,271.0	2584.0	2648.0
7	7	3 (0)	n.a.	t1743 (1)	0	2,747,478.7	66.0	147,717.0	2521.3	2588.0
8	8	11 (6)	IV (6)	t008 (6)	5	2,821,267.1	52.7	259,331.5	2625.2	2681.8
293		2 (2)	V (1)	t037 (1)	0	2,900,431.5	90.5	90,036.5	2697.5	2762.0
241		1 (1)	n.d.	t030 (1)	0	2,884,707.0	87.0	105,325.0	2707.0	2768.0
247		1 (1)	I (1)	t197 (1)	0	2,776,359.0	76.0	74,230.0	2567.0	2630.0
10	10	1 (0)	n.a.	n.a.	0	2,799,287.0	110.0	52,819.0	2634.0	2698.0
1162		2 (0)	n.a.	n.a.	0	2,867,105.0	58.0	184,821.5	2702.0	2767.0
15	15	5 (2)	IV (1); I (1)	t084 (1); t853 (1)	1	2,719,481.8	45.6	226,394.0	2496.2	2556.6
22	22	15 (14)	IV (13)	t852 (1); t1977 (7); t223 (1); t005 (1)	3	2,793,443.3	56.0	124,918.3	2599.5	2662.4
1327		1 (1)	IV (1)	n.a.	0	2,758,892.0	42.0	167,290.0	2547.0	2612.0
25	25	2 (0)	n.a.	t258 (1); t2242 (1)	0	2,758,786.5	16.5	697,459.0	2554.5	2617.5
30	30	7 (3)	IV (3)	t019 (2)	5	2,792,108.9	58.1	139,913.6	2603.3	2666.1
34		2 (0)	n.a.	t3905 (1)	0	2,821,562.0	57.5	140,057.0	2665.5	2730.5
45	45	8 (2)	IV (2)	t015 (2)	0	2,762,203.4	34.4	390,455.1	2591.5	2654.6
59	59	3 (3)	IV (1)	t216 (1); t437 (1)	1	2,799,567.0	53.0	130,817.3	2595.7	2662.0
88	88	1 (1)	IV (1)	t4701 (1)	0	2,791,324.0	36.0	206,283.0	2575.0	2642.0
96	96	1 (0)	n.a.	n.a.	1	2,783,146.0	39.0	141,877.0	2591.0	2652.0
97	97	2 (2)	IV (2)	t359 (1)	0	2,756,222.0	27.0	401,302.0	2570.0	2635.5
121	121	12 (0)	n.a.	t3274 (1); t314 (1); t2530 (1)	9	2,814,764.5	48.0	146,041.3	2631.3	2694.3
152	152	3 (2)	V (2)	t355 (1)	2	2,753,826.7	31.0	267,208.7	2551.0	2608.0
395	395	1 (0)	n.a.	n.a.	0	2,759,659.0	22.0	574,657.0	2574.0	2640.0
398	398	2 (1)	V (1)	t011 (1)	0	27,540,120.0	57.5	20,645,930	2524.0	2589.5
942	942	1 (0)	n.a.	n.a.	0	2,813,978.0	82.0	61,174.0	2654.0	2718.0
–	n.a.	3 (1)	IV (1)	n.a.	0	2,739,763.7	46.0	157,754.0	2535.0	2599.3

The combination of the four methods (MLST, SCC*mec*-, and *spa*-typing, and PVL presence) yielded 80 different lineages. Three isolates were not assigned to any specific ST and are reported in the last row of the table

According to both antibiotic susceptibility testing (oxacillin and cefoxitin susceptibility, Additional file 1: Table S1) and genome analysis (presence of the SCC*mec* cassette, see Materials and methods), 63.7% of the isolates were classified as methicillin-resistant *S. aureus* (MRSA). Most strains (*n* = 54) belonged to SCC*mec*IV; type I cassettes were also abundant (*n* = 19), whereas cassettes type V (*n* = 8) and II (*n* = 1) were less represented. Methicillin resistance was unevenly distributed across the phylogenetic tree (Fig. 1) and partially independent from the STs. All CC1 isolates (*n* = 14, ST1 and ST772) were MRSA, and so were the isolates belonging to CC5 (*n* = 30, ST5 and ST228) and CC22 (*n* = 16, ST22 and ST1327). All CC121 (*n* = 12, ST121) and CC10 (*n* = 3, ST10 and ST1162) isolates were instead methicillin-sensitive (MSSA), and other clonal complexes (CC8, CC30, CC45) showed balanced proportions of sensitive and resistant strains. SCC*mec*I (*n* = 19) was the most CC-specific cassette, as it was found almost exclusively in CC5 isolates (ST5 and ST228), with the exception of one ST15 and one ST8 isolates, while neither SCC*mec*IV nor SCC*mec*V were associated with specific STs.

For five of the recovered STs, namely ST241, ST942, ST1162, ST1327, and ST1866, no sequenced genome is publicly available (as genomes of *S. aureus* in RefSeq [88] version 2017 [89]). Although a large number of *S. aureus* genome sequences are available in NCBI, these are biased toward a limited set of clinically relevant STs [43, 90], with many others being neglected. This underrepresentation of less-pathogenic or less-known strains may lead to a poor understanding of the host–pathogen interactions at the genomic level, and to an underestimation of emerging or re-emerging pathogenic strains [25, 43].

### Co-presence of local, global, animal-associated, and hypervirulent clones

We combined the four characterisation methods (MLST, SCC*mec*-, *spa*-, and PVL typing) to identify specific known clones in the cohort, yielding 80 different lineages. The most prevalent were the South German/Italian ST228-SCC*mec*I clone (*n* = 16, 11.85%) and the E-MRSA-15 ST22-SCC*mec*IV clone (*n* = 13, 9.63%), followed by the USA400 ST1-SCC*mec*IV t127 (*n* = 11, 8.15%) clone, the USA800 paediatric clone ST5-SCC*mec*IV t002 (*n* = 10, 7.41%), and the USA500 E-MRSA-2/6 clone ST8-SCC*mec*IV t008 PVL− (*n* = 4, 2.96%) (Additional file 5: Table S4). Several other clones, including the highly virulent USA300 ST8-SCC*mec*IV PVL+ clone (*n* = 2, 1.48%), were also found, confirming a heterogeneous clone composition in Italian hospitals [91, 92]. Surprisingly, we did not isolate any ST80, the most prevalent community-associated MRSA clone in Europe [93].

We moreover identified two isolates (1.48%) belonging to the livestock-associated MRSA (LA-MRSA) ST398 clone [24, 94] (Table 1). This clone has already been reported in patients that had regular exposure to livestock in several countries [24, 95, 96] including Italy [97–99], but our results and other reports [96, 100–102] of infections in non-exposed subjects suggest that the within-subject transmission for these clones is not rare. Similar conclusions can be drawn for another LA-MRSA, namely ST97 (*n* = 2, 1.48%, Table 1), which is the leading cause of bovine mastitis, but is only rarely reported in humans [103–106]. This growing incidence of LA-MRSA strains (*n* = 4, 2.96% in our cohort) causing zoonotic infections highlights the existence of underestimated reservoirs of *S. aureus* strains that could become epidemic [28, 107, 108].

One isolate was assigned to ST395, which is an unusual strain unable to exchange DNA via bacteriophages with other *S. aureus* strains because of a modification in the wall teichoic acid (WTA) [109, 110]. The same modification, however, enables ST395 to exchange DNA with coagulase-negative staphylococci (CoNS) [110], making it particularly prone to exchange SCC*mec* elements and others with other commonly found staphylococci, e.g. *S. epidermidis*.

### Genomic signatures of chronic versus acute *S. aureus* infections

In order to investigate the potential association of clones and antibiotic resistance with specific hospital operative units, we cross-checked the prevalence of SCC*mec* types, STs, and PVL+ clones with both OUs and sample types (see Materials and methods). Strains from the cystic fibrosis (CF, *n* = 76) unit were positively associated with the presence of SCC*mec*I (*n* = 19, ten from CF unit; *p* value = 0.03), a cassette known to be hospital-associated [111, 112]. Strains from the same unit were also associated with ST1 (*n* = 12, seven from CF unit; *p* value = 0.04), whereas we noted a reduced prevalence of the PVL genes (*n* = 37, only two from CF unit; *p* value = 0.0002) and of ST121 (*n* = 12, none from CF unit; *p* value = 0.02). This reflects the relatively attenuated virulence which is a well-known phenomenon in long-term *S. aureus* infections [113–116]. Similarly, sputum samples (*n* = 33; 88.7% from CF unit) were associated with ST228 (*n* = 16, nine from sputum; *p* value = 0.004) and SCC*mec*I (*n* = 19, 11 from sputum; *p* value = 0.0008), and negatively correlated with PVL (*n* = 37, only two from sputum; *p* value = 0.001). The high correlation of ST228 with lung isolates and specifically with CF has already been observed in Spain [111]. A similar pattern of increased resistance and lowered virulence has been observed for another sample type linked with long-term lung infections, namely broncho-aspiration material (*n* = 23; 78.2% from intensive care unit). Strains from this sample type were associated to SCC*mec*IV (*n* = 54, 14 from broncho-aspiration material; *p* value = 0.008), and with PVL− (*n* = 98, 23 from broncho-aspiration material; *p* value = 0.0005) and MRSA clones (*n* = 83, 21 from broncho-aspiration material; *p* value = 0.002), highlighting once again the loss of virulence and the acquisition of resistance in long-term lung infections [113–116].

On the contrary, patients from both emergency room (*n* = 5) and the infectious disease unit (*n* = 15) show an overrepresentation of PVL+ clones (*n* = 37, four from emergency room and nine from infectious diseases; *p* values = 0.02 and 0.005, respectively), indicative of acute rather than chronic infections. Lesion swabs (*n* = 31) are strongly associated with MSSA (*n* = 49, 31 from lesion swabs; *p* value = 3e−08). This sample type was also associated to the hypervirulent ST121 clone [117, 118] (*n* = 12, 11 from lesion swabs; *p* value = 2e−05) and to the presence of the PVL (*n* = 37, 14 from lesion swabs; *p* value = 3e−07), suggesting that in our cohort skin and soft tissue infections (SSTIs) are predominantly caused by hypervirulent MSSA strains. Lesion swabs from children in care at the infectious diseases unit (*n* = 12, 80% of the samples from this operative unit) are also characterised by high prevalence of the virulent ST45 clone [119, 120] (*n* = 8, three from lesion swabs; *p* value = 0.04) that is known to be associated with SSTIs [121–124]. The expected [125] association between PVL (*n* = 37) and ST121 (*n* = 12, nine PVL+; *p* value = 0.001) and ST30 (*n* = 7, five PVL+; *p* value = 0.003) supports once again the observed increased virulence of these STs [117, 118, 126, 127], which is partially in conflict with the hypothesis of lesion colonisation by commensal strains present in the skin microbiome [128, 129].

### Discovery of novel variants of SCC*mec*IV with kanamycin, trimethoprim, and bleomycin resistance

We next investigated the specific genetic variants of the four types of SCC*mec* cassettes identified and discussed above. This is relevant because the epidemiology of this genetic element is disentangled by that of the rest of the genome by virtue of its high horizontal mobility [130, 131]. Moreover, the SCC*mec* can host genes encoding not only for resistance to beta-lactams [132, 133], but also for other antibiotic resistances or virulence factors [131].

More than a half of the MRSA isolates in our collection (*n* = 86) carried SCC*mec*IV (62.8%). This cassette type has spread widely in the last decades, often substituting the previously more prevalent nosocomial SCC*mec* types I and II [24, 134], and it is now common especially in European clinical isolates [24, 92]. Another cassette that has spread in recent years following a similar path is SCC*mec*V [134, 135], the third most prevalent cassette type in our cohort (10.5% of the MRSA isolates) after the more traditionally hospital-associated SCC*mec*I [24, 112] (22.1% of the MRSA isolates). We moreover isolated one MRSA carrying SCC*mec*II, which is widely diffused in the USA but only rarely found in Italy/Europe [25, 136]. Consistently, the SCC*mec*II isolate was recovered from Patient 115, which is consistent with the personal history of the patient. For two isolates, it was not possible to classify the cassette neither with PCR nor with in silico PCR using standard primers [45].

**Fig. 3:**
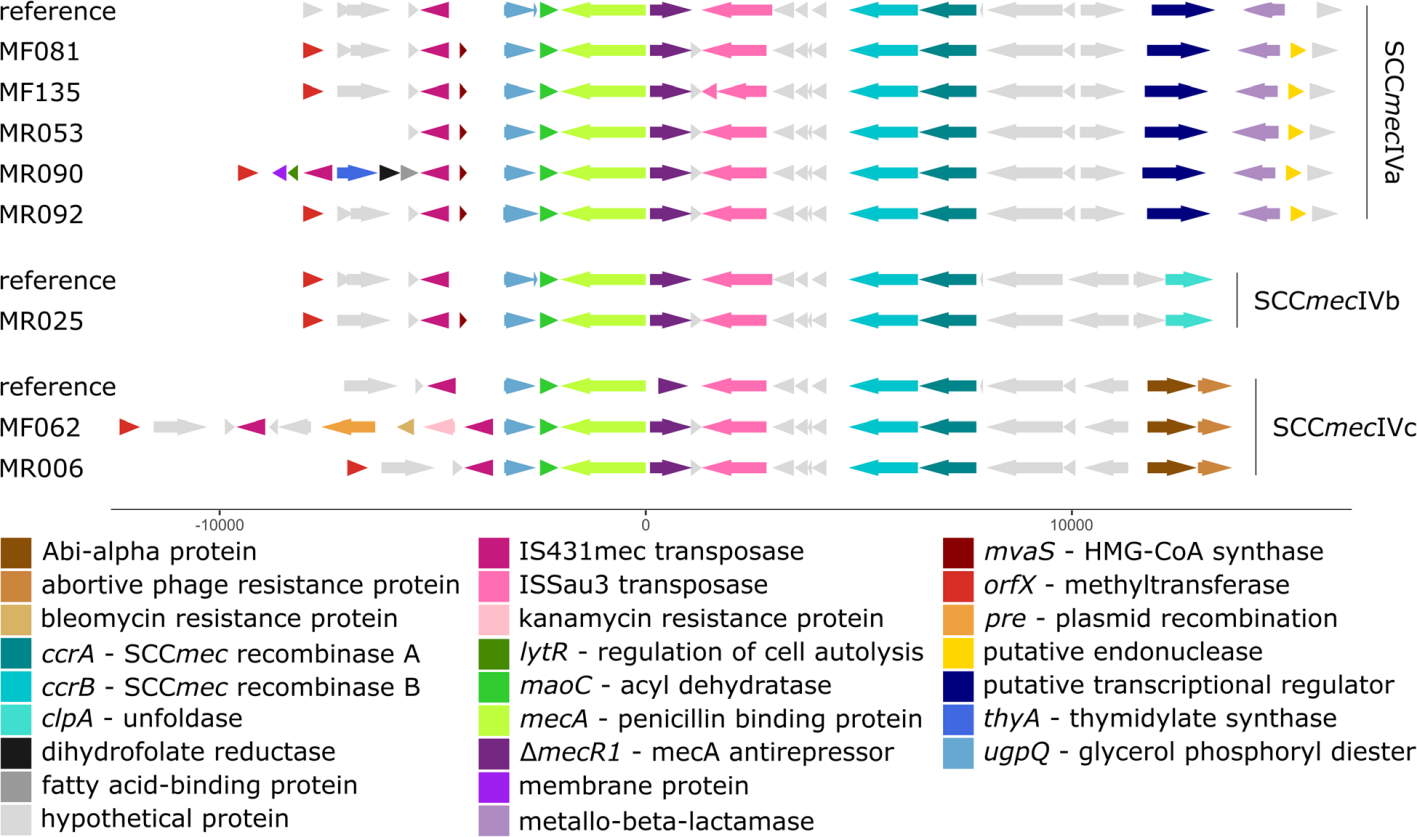
Overview of the SCC*mec*IV cassette variability in our cohort, compared with available reference cassettes for the recovered subtypes IVa, IVb, and IVc. Genes are marked as arrows in the direction of transcription. To avoid biases due to misassemble of the region of interest, only cassettes found on a single contig are reported. Annotated SCC*mec* are grouped together with the closest reference cassette subtype. Some genomes showed insertions of genes involved in resistance to trimethoprim (MR090) and to kanamycin and bleomycin (MF062)

By aligning reconstructed SCC*mec* with reference cassettes (see Materials and methods), we observed a certain degree of variability inside the same cassette type, specifically in type IV (Fig. 3). Subtypes IVa, IVb, and IVc were identified, with some SCC*mec* elements showing insertions. Two cassettes in particular were not consistent with the already described subtypes: the SCC*mec* type IVc carried by MF062, which was enriched with genes for kanamycin [137] and bleomycin [138, 139] resistance, and the type IVa carried by MR090 that showed insertion of genes involved in resistance to trimethoprim [140, 141] (Fig. 3).

### Non-SCC*mec* resistance profiles show different patterns in chronic and acute infections

**Fig. 4:**
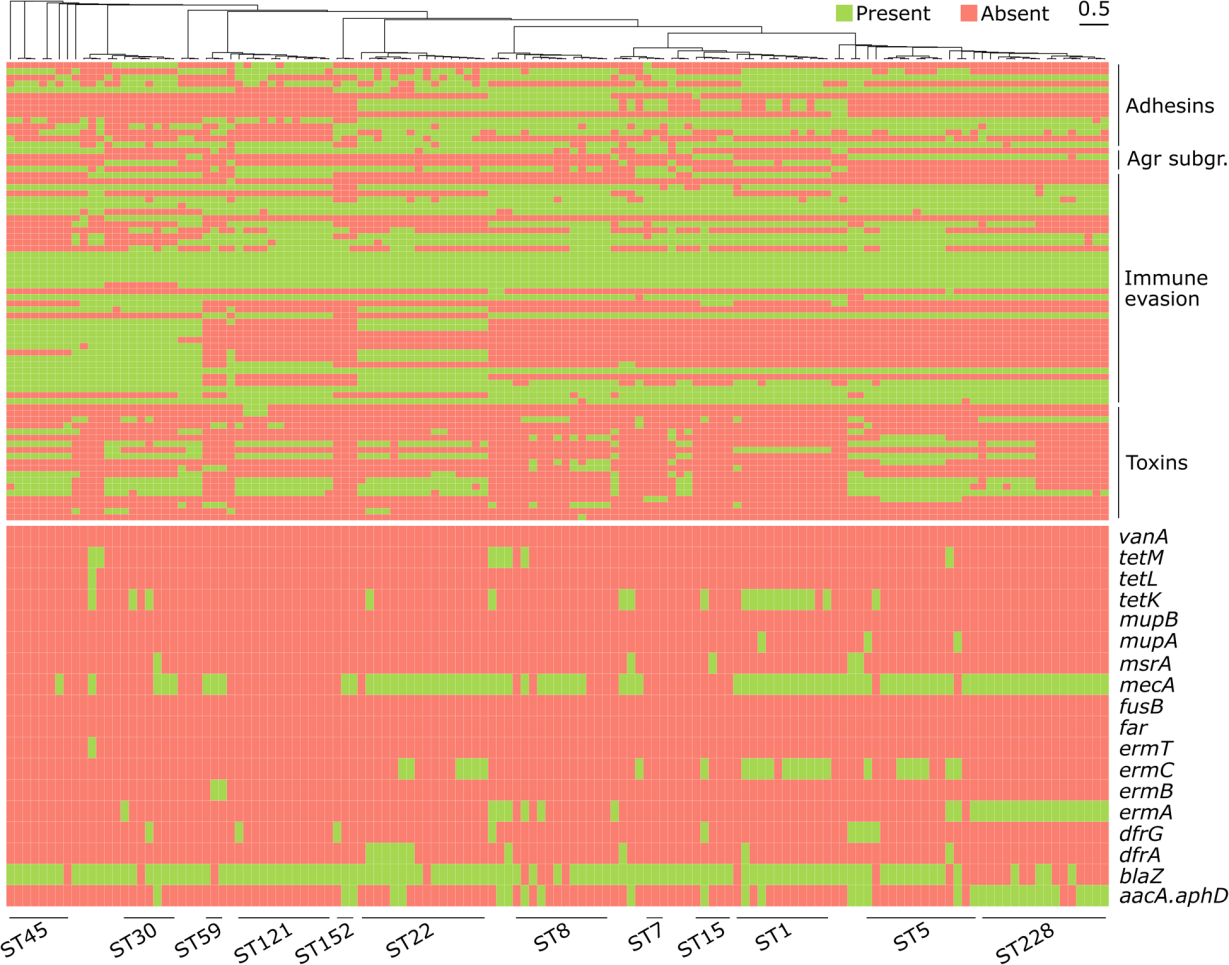
Presence/absence profile of 79 genes encoding for virulence factors (upper part of the heatmap) and 18 genes encoding for resistance (bottom part). Some virulence and resistance factors were more represented in specific STs (only STs found in > 1 samples are specifically mentioned), as in the case of gentamicin resistance that is more prevalent in the ST228 isolates. For a more detailed overview of the single genomes’ profiles, see Additional file 3: Table S2

*S. aureus* can easily acquire a number of resistances, including those to the last resort antibiotics vancomycin [142, 143] and daptomycin [144]. According to results presented in previous paragraphs and elsewhere [145], resistances can occur by gene acquisition in the SCC*mec* cassette. Most resistances are however encoded by genes that are found in other parts of the genome or that have been horizontally transferred through different genetic elements [25]. Given the high importance of multi-drug resistance in *S. aureus* [20], we therefore tested the presence or absence of specific resistance genes in our cohort [146] (Fig. 4 and Additional file 3: Table S2). Consistently with previous literature [6], most of the isolates tested positive for *blaZ* (81.5%), responsible for penicillin resistance (96.3% concordance with antibiotic susceptibility test, as per presence of the *pbp* and/or *mecA* genes). No isolates were found positive for genes encoding resistance to vancomycin (*van*, 100% concordance with antibiotic susceptibility test) and to fusidic acid (*fusB* and *far*, 94.1% concordance with antibiotic susceptibility test). Antibiotic resistances were sometimes associated with specific CCs, as for the increased representation of *aacA.aphD* (gentamicin resistance, 92.6% concordance with antibiotic susceptibility test) and *ermA* (erythromycin resistance, phenotypic resistance not tested) in CC5 isolates, whose genomes tended to lack instead the *blaZ* gene (penicillin resistance) (Fig. 4). Overall, two isolates from acute skin infections were negative for all the resistance genes tested, while six CF and intensive care unit isolates were positive for six (33.3%) of them. This pattern of increased resistance in long-term infections, together with their observed reduced virulence, completes the scenario of reduced virulence and increased resistance that has been observed in this and previous studies [113–116].

### Emergence and disease-associated diversity of clinically relevant virulence factors

*S. aureus* has a large repertoire of virulence genes, and it is able to evade the host immune system through a variety of strategies. Some of the genes usually involved in immune evasion were present in almost all our isolates (Fig. 4 and Additional file 3: Table S2). These include genes encoding the phenol-soluble modulin alpha and beta and the delta-haemolysin Hld, responsible for leukocytes and erythrocyte lysis respectively [60]; the immunoglobulin-binding protein Sbi that inhibits IgG and IgA [61, 62]; and some genes part of the GIɑ genomic island (*ssl6* and *ssl9*).

Other genes belonging to the immune evasion island IEC2 were present in many but not all isolates, for example, the one encoding for the antiplatelet extracellular fibrinogen binding protein Efb [63, 64] and those encoding various haemolysins (*hla*, *hlg*) [59, 60] (Fig. 4 and Additional file 3: Table S2). In addition to the 27.4% prevalence of the *lukF* and *lukS* PVL genes discussed above, one sample (MR029, from emergency room) was positive for the epidermal cell differentiation inhibitor Edin, which has been found to promote the translocation of *S. aureus* into the bloodstream [65]. One of the two USA300 isolates (MR047, from nasal swab) tested positive for the arginine catabolic mobile element (ACME), another important virulence factor (gene *arcA*) that has been shown to be responsible for the increased pathogenicity of *S. aureus* and specifically of USA300 clones [66, 67].

Many virulence genes were associated to specific STs (Fig. 4 and Additional file 3: Table S2). ST22 (*n* = 15), for instance, was associated with the toxic shock syndrome toxin TSST-1 (*n* = 8, three from ST22; *p* value = 0.04; present in 20% of the ST22 clones) [60, 68, 69], other pyrogenic toxin superantigens known as staphylococcal enterotoxins (SEs, mean *n* = 34.9 ± 28.1 s.d.; *p* value < 0.02 for *seg*, *sei*, *sem*, *sen*, *seo*, present on average in 86.7% of ST22 and 49.5% of non-ST22), and various *ssl* immune evasion genes (mean *n* = 57.4 ± 40.2 s.d.; *p* value < 0.01 for *ssl1*, *ssl3*, *ssl4*, *ssl7*, *ssl11*, *ssl12*, present on average in 93.3% of ST22 and 21.1% of non-ST22) [70, 71]. ST22-IV EMRSA-15 clones positive for *tst1* are usually described as “Middle Eastern variant” [72–74], but a high prevalence in an Italian neonatal intensive care unit [76] and pre-school children living in Palermo, Italy [75], has been observed. Authors suggested that the Middle Eastern clone might be more widely spread than estimated and might have diffused in the Mediterranean populations as a community-acquired MRSA [75, 76], as suggested by our analysis. TSST-1 is responsible for an increased pyrogenic, emetic, and superantigen activity, together with SEs (10627489; 11544350). SEs (mean *n* = 34.9 ± 28.1 s.d.) were associated with all “virulent” STs, such as ST5 (*n* = 15, *p* value < 0.04 for *sed*, *seg*, *sei*, *sej*, *sem*, *sen*, *seo*, *sep*, present on average in 79.2% of ST5 and 32.3% of non-ST5), ST45 (*n* = 8; *p* value < 0.02 for *sec*, *seg*, *sei*, *sel*, *sem*, *seo*, present on average in 98.2% of ST45 and 38.9% of non-ST45), ST121 (*n* = 12; *p* value < 0.02 for *seb*, *seg*, *sei*, *sem*, *sen*, *seo*, present on average in 86.1% of ST121 and 41.7% of non-ST121), and—to a lower extent—ST30 (*n* = 7; *p* value < 0.02 for *sei*, *sem*, *sen*, present on average in 100% of ST30 and 50% of non-ST30).

The hypervirulent ST121 MSSA isolates obtained from lesion swabs (*n* = 12) were instead associated with the genes encoding for the exfoliative toxins Eta and Etb (*n* = 3 from ST121 swabs, and *n* = 0 for non-ST121, *p* value = 0.0006 for both genes), responsible for the skin manifestations of bullous impetigo and Staphylococcal scalded skin syndrome [77–79], the gene *bbp* (*n* = 12 from ST121, *n* = 7 from non-ST121; *p* value = 1.35e−08) that interacts with the extracellular matrix bone sialoprotein and contributes to staphylococcal arthritis and osteomyelitis [80], and the immune evasion gene *ecb* (*n* = 12 from ST121, *n* = 36 from non-ST121; *p* value = 1.51e06), which is required for the persistence of *S. aureus* in host tissues and the formation of abscesses [81]. The latter was also present in all and only the isolates belonging to ST1, ST7, ST10, ST15, ST30, ST34, and ST398, suggesting a strong dependence on ST (Fig. 4 and Additional file 3: Table S2).

Isolates retrieved from sputum samples of CF patients (*n* = 38) showed a positive association with the adhesin-encoding genes *sdrD* (*n* = 34 from CF, *n* = 69 from non-CF; *p* value = 0.03) and *sdrE* (*n* = 27 from CF, *n* = 48 from non-CF; *p* value = 0.03), and a negative association with *bbp* (*n* = 1 from CF, *n* = 18 from non-CF; *p* value = 0.01), contrary to samples from the infectious disease unit (*n* = 15, four positive for *bbp* gene). This finding is consistent with the increased need for adhesins in chronic lung infections [82, 83, 116], including in CF [84].

### Conservation of genes encoding vaccine candidates

Unlike other bacterial infections, prior exposure to *S. aureus* does not seem to provide protective immunity [147]; therefore, vaccines are an attractive yet challenging option to prevent disease. Researchers have long attempted to produce an effective vaccine against *S. aureus*, but even though few have proved promising in animal models, the two vaccines so far tested in efficacy clinical trials have failed [147–153]. Since the main issue is the polymorphic expression of *S. aureus* surface antigens and the redundancy of its virulence proteins [147, 154, 155], we tested the prevalence and conservation of a number of genes encoding vaccine candidates described in the literature (Table 2).

**Table 2:** Sequence variability of genes of interest for vaccine development. Number (and relative abundance) of isolates positive for the gene, followed by the percentage of positive isolates carrying 0 or less than 1%, 2%, 5%, or more/equal to 5% of non-synonymous SNVs or insertions-deletions (indels) with respect to reference gene. Both clinical trial IDs (ClinicalTrials.gov database identifiers, http://clinicaltrials.gov) and reference studies refer to the latest available trials

Gene	# positive isolates (%)	Distribution of non-syn SNVs w.r.t. reference seq.						Latest trials	ClinicalTrials identifier	Ref.
		0	< 1%	< 2%	< 5%	≥ 5%	indels			
*clfA*	95 (70.4%)	0%	9.5%	0%	0%	0%	90.5%	Phases I–II	NCT01643941; NCT01364571	[159, 160, 171]
*csa1a*	70 (51.9%)	41.4%	4.3%	0%	0%	0%	54.3%	Preclinical		[168, 172]
*csa1b*	36 (26.7%)	19.4%	47.2%	0%	2.8%	0%	30.6%			
*esxA*	134 (99.3%)	85.1%	14.9%	0%	0%	0%	0%	Preclinical		[168, 172]
*esxB*	89 (65.9%)	0%	98.9%	1.1%	0%	0%	0%	Preclinical		[168, 172]
*esxC*	89 (65.9%)	25.8%	40.4%	33.7%	0%	0%	0%			
*esxD*	89 (65.9%)	58.4%	41.6%	0%	0%	0%	0%			
*fhuD2*	135 (100%)	31.1%	68.9%	0%	0%	0%	0%	Preclinical		[168, 172]
*hla*	124 (91.9%)	6.5%	0%	9.7%	78.2%	2.4%	3.2%	Phase II	NCT02296320	[168, 172, 173]
*hlgA*	135 (100%)	65.2%	29.6%	2.2%	0%	0%	3%			
*hlgB*	132 (97.8%)	11.4%	65.2%	22.7%	0%	0%	0.8%	Preclinical		[174]
*hlgC*	135 (100%)	61.5%	8.1%	28.1%	2.2%	0%	0%			
*isdB*	134 (99.3%)	11.9%	21.6%	0%	0%	0%	66.4%	Phase III	NCT00518687	[152, 166]
*lukF*	37 (27.4%)	0%	97.3%	0%	0%	0%	2.7%			
*lukS*	37 (27.4%)	56.8%	40.5%	0%	0%	0%	2.7%	Phases I–II	NCT01011335	[175]
*mntC*	135 (100%)	79.3%	20.7%	0%	0%	0%	0%	Phases I–II	NCT01643941; NCT01364571	[159, 160, 171]
*tst*	8 (5.9%)	0%	100%	0%	0%	0%	0%	Phase I	NCT02340338	[176]

Among antigens that have been proposed as targets for vaccine development, the alpha haemolysin toxin gene *hla* [147, 156, 157] and the genes coding for capsular biosynthesis *cap5* and *cap8* [150, 151] are highly prevalent in our cohort (91.9% and 97.8% of the isolates respectively). Nevertheless, these genes showed a larger degree of variability compared to the others we considered, which may explain the poor results obtained in clinical trials [147, 150, 151, 156, 157]. Other genes that code for proteins used alone or in combination in vaccine formulations, such as the virulence determinant SpA [158] and the fibronectin-binding protein ClfA [159–161], are present in most of our strain collection. In some of these genes, indels are prevalent (> 90%, Table 2), but they are frequently found in repeated regions that may not critically impact the protein structure, as in the case of the *spa* gene.

Vaccines have also been proposed for *S. aureus* strains with specific characteristics. For instance, targeting the toxicity determinant TSST-1 (5.9% prevalence of *tst1*) [162, 163] or the PVL proteins LukF-LukS (27.4% prevalence of *lukF-lukS*) [164, 165] aims at selectively preventing the most virulent or lethal infections. In our cohort, despite their low prevalence, both *tst1* and the PVL genes were conserved at 99%, except for a few isolates that had indels in the latter (Table 2). The gamma-haemolysins HlgAB and HlgCB genes [164, 165] were instead highly prevalent (97.8–100%) and quite conserved (69.6–94.8%). The opposite approach is targeting genes with a lower virulence profile, which may be more prevalent and conserved than those coding for highly toxic factors. Among them, the genes encoding for the manganese uptake receptor (*mntC*) [159–161] and for the iron acquisition factor (*isdB*) [152, 166], which are indeed present in all or all but one the isolates of our cohort. Non-synonymous mutations are rare in *mntC* (20.7% of the isolates, with only one non-synonymous SNV), and, whenever not affected by indels that may or may not affect the protein structure, also the *isdB* gene is highly conserved (> 99% identity, Table 2).

Finally, we also analysed the conservation of *csa1A*, *csa1B*, *fhuD2*, and *esxA*, genes recently described as being promising vaccine candidates in preclinical studies [167, 168]. The two genes encoding for the conserved antigen Csa (*csa1A* and *csa1B*) are present in 51.9% and 26.7% of the isolates respectively and are conserved in only a fraction of the cases (Table 2). By contrast, the iron uptake gene *fhuD2* is present in all isolates, with a maximum of 1% non-synonymous variation in sequence (Table 2). Also the genes encoding for the ESAT-6-like secretion system (*esxA*, *esxB*, *esxC*, *esxD*) are well represented in the cohort, but only *esxA* is present in all but one isolate and has no non-synonymous mutations in 85.1% of the isolates (Table 2). Therefore, on the basis of their conservation, both FhuD2 and EsxA appear to be promising targets for vaccine formulations.

### Phylogenetics of specific STs highlights the aggressive spread of a novel independently acquired ST1 clone

We investigated the hypothesis that some of the prevalent STs could be hospital-associated clones. We estimated the ST phylogenies using a whole-genome maximum likelihood approach (see Materials and methods). In most cases, we observed that isolates in our cohort, despite sharing the same ST, SCC*mec*, and *spa* types, were not monophyletic subtrees when considering external reference genomes for the same STs. This is the case, for example, of the ST228 and ST5 clones (Fig. 2). This suggests independent acquisition of the clones and no evidence of transmission among the selected hospitalised patients, while person-to-person transmission from healthy carriers or non-selected patients cannot be ruled out [21, 22]. Only two ST121 MSSA isolates were found to be almost identical and both were retrieved in the same time window from patients 096 and 098 (8 SNVs). For ST1, instead, all but two isolates belonged to the same sub-lineage, typed as SCC*mec*IV t127 PVL−.

**Fig. 5:**
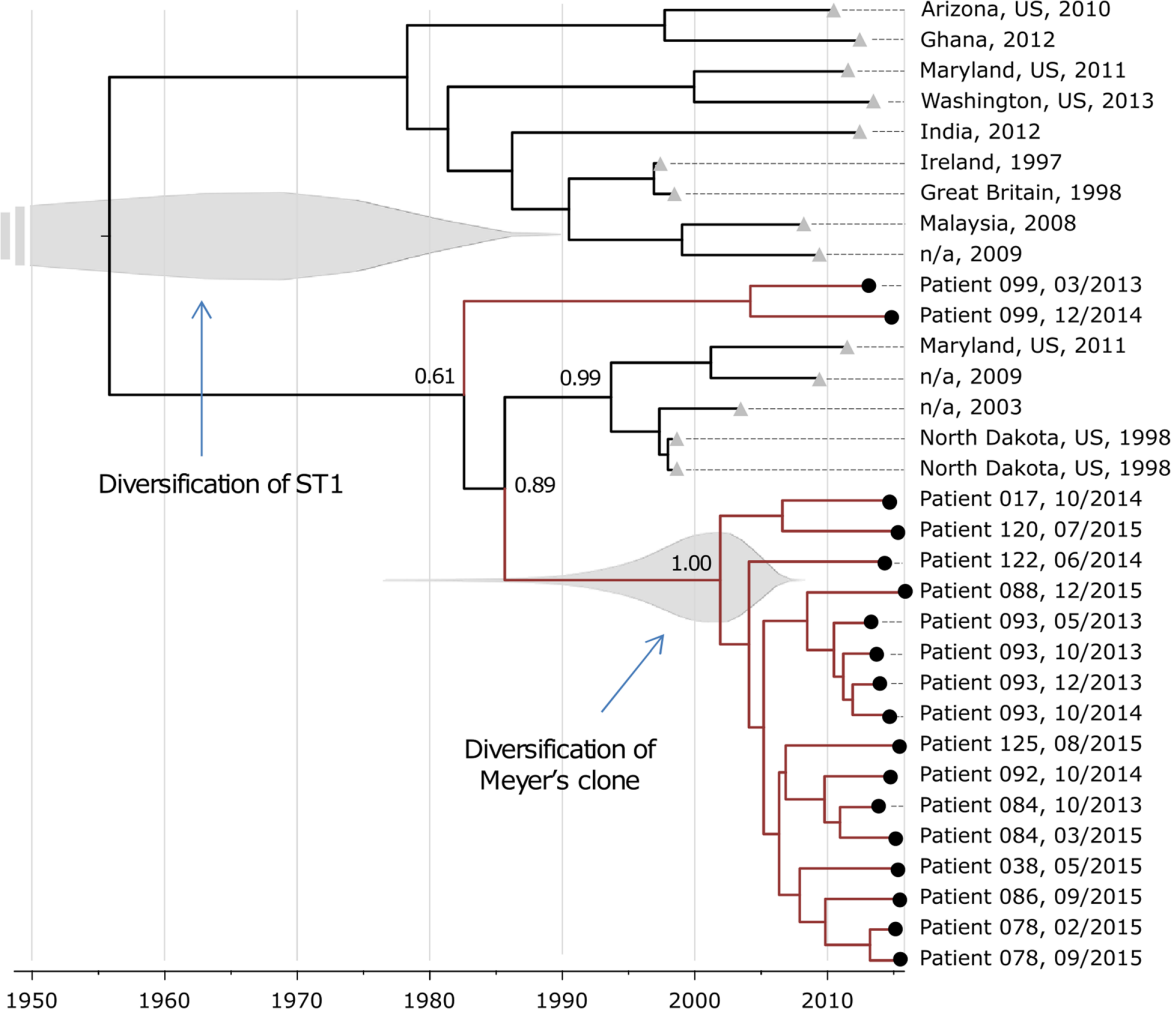
Bayesian timed tree of ST1 isolates, including reference genomes. Location and date of sample collection is reported for each isolate. For samples collected at Meyer’s Children Hospital (black circles), patient code is reported instead of location. The two North Dakota samples were collected from the same subject. “n/a” indicates that no information is available for location of sample collection. Numbers at selected nodes are posterior probabilities. Grey areas are the distributions of marginal posterior probabilities for the diversification of ST1 and the diversification of Mayer-specific clone

We further estimated divergence times for all the 16 isolates belonging to the ST1 SCC*mec*IV t127 PVL− clone, including those obtained from earlier or later time points of the same patients. We used a Bayesian approach [85] (see Materials and methods) integrating all the reference genomes publicly available for ST1 and the two ST1 SCC*mec*V isolates from our cohort (Additional file 4: Table S3). These analyses were performed to test the hypothesis that all ST1 SCC*mec*IV t127 belong to a clone specific of Meyer’s hospital. The relaxed exponential clock model with constant coalescent prior and GTR substitution model resulted to the most appropriate model (Additional file 6: Table S5). This model estimated that Meyer’s clone has emerged approximately 6 to 28 years ago as a specific branch of the ST1 tree, which has been estimated to be 26–160 years old (Fig. 5). However, age of Meyer’s clone does not match with the time of emergence of the clone in the hospital. Moreover, an isolate obtained in a recent study investigating the spread of a ST1 SCC*mec*IV t127 clone in Irish hospitals [169] and carrying a virulence and resistance profile very close to the one of our cohort (differences in gene presence: 2/79 and 0/18 respectively) is phylogenetically rooted inside Meyer’s cluster (161 SNVs intra-cluster; 412 SNVs inter-cluster). These two findings suggest that ST1 SCC*mec*IV t127 is not specific of the Meyer Children’s hospital but might represent a newly arising community clone that is now spreading in the nosocomial environment of different countries [169, 170].

## Conclusions

In this study, we investigated the epidemiology of *S. aureus* in different operative units of Anne Meyer’s Children’s University Hospital (Florence, Italy) over a timespan of 3 years by whole genome isolate sequencing. Our analyses highlighted a high diversity of STs, SCC*mec*, and *spa*-types, resulting into a wide number of clones. Some of these clones had been previously described in the literature as livestock-associated, and we described them in non-exposed children thus supporting the spreading of such clones in the non-at-risk community. We moreover described the presence of hypervirulent and geographically unusual clones, and of five STs for which no sequenced genome was available in public databases. Our refined analysis of the SCC*mec* cassettes highlighted the presence of further resistances and diversity within the same cassette type. On the contrary, when considering single infection types or specific STs or clones as it is usual in *S. aureus* epidemiological studies, the genomic diversity was limited, with an increased pattern of resistance genes in chronic patients and a larger number of virulence factors in acute infections. Altogether, these observations shed more light on the complexity of *S. aureus* epidemiology and on the need for a more unbiased survey of the commensal and pathogenic *S. aureus* community, to avoid the misrepresentation of specific genomic traits.

Whole-genome-based routine surveillance of *S. aureus* and other hospital-related pathogens would further allow to get a more unbiased idea of the rising clones and better informing clinical practices, which usually focused on the most dangerous or well-known strains. Performing such epidemiological studies as soon as a new putative nosocomial clone arises could allow us to conclude whether the new clone has arisen in that very hospital or it is a recent sub-clone spreading also in the non-hospitalised population and therefore more frequently isolated also in the clinics. These wider-focus studies would not only allow the assessment of the epidemiology of specific pathogens and clones in the hospital setting, but also the survey of the prevalence and conservation of their virulence and resistance traits. This could lead to the identification of antigens of interest for vaccine development and of specific sub-clones representing the main burden of infection, and therefore reassessing the efforts for the discovery of new treatments.

Whole genome sequencing studies are crucial to survey the global epidemiology of infectious agents, including *S. aureus*, as genome-based data are reproducible and can be easily meta-analysed without the confounding of batch effects. The meta-analysis of pathogenic, commensal, and environmental *S. aureus* isolates could lead to a deeper knowledge of the epidemiology of this bacterium and may help in understanding how to prevent and treat infections without boosting antibiotic resistance.

## Additional files

Additional file 1: **Table S1.** Characteristics of the single isolates, including collection details, genome assembly statistics, genomic features, and results of antibiotic susceptibility testing. (XLSX 69 kb)

Additional file 2: **Figure S1.** Pangenome analysis statistics. **Figure S2.** Phylogenetic model based on gene presence/absence. (PDF 580 kb)

Additional file 3: **Table S2.** Presence/absence profile of virulence and antibiotic resistance genes in the cohort. (XLSX 6063 kb)

Additional file 4: **Table S3.** Characteristics of the genomes included in the ST1 analyses. (XLSX 11 kb)

Additional file 5: **Table S4.** Most relevant clones represented in the cohort and their abundance. (XLSX 9 kb)

Additional file 6: **Table S5.** Bayesian clock models tested and their results. (XLSX 10 kb)

## Abbreviations

CC: Clonal complex
CDS: Coding sequence
CF: Cystic fibrosis
CoNS: Coagulase-negative staphylococci
indels: Insertions and deletions
MLST: Multilocus sequence typing
MRSA: Methicillin-resistant *S. aureus*
MSSA: Methicillin-sensitive *S. aureus*
PVL: Panton-Valentine Leukocidin
SCCmec: Staphylococcal Cassette Chromosome *mec*
SNV: Single-nucleotide variation
Spa: Staphylococcal protein A
ST: Sequence type
WGS: Whole-genome shotgun sequencing
