# Supplementary material for: Whole-genome epidemiology, characterisation, and phylogenetic reconstruction of Staphylococcus aureus strains in a paediatric hospital
Source: Genome Med. 2018 Nov 13;10:82. doi: 10.1186/s13073-018-0593-7 (PMC6234625; doi:10.1186/s13073-018-0593-7)
Supplement: Supplementary file 2 — Figure S1. Pangenome analysis statistics. Figure S2. Phylogenetic model based on gene presence/absence. (PDF 580 kb) [file 13073_2018_593_MOESM2_ESM.pdf]

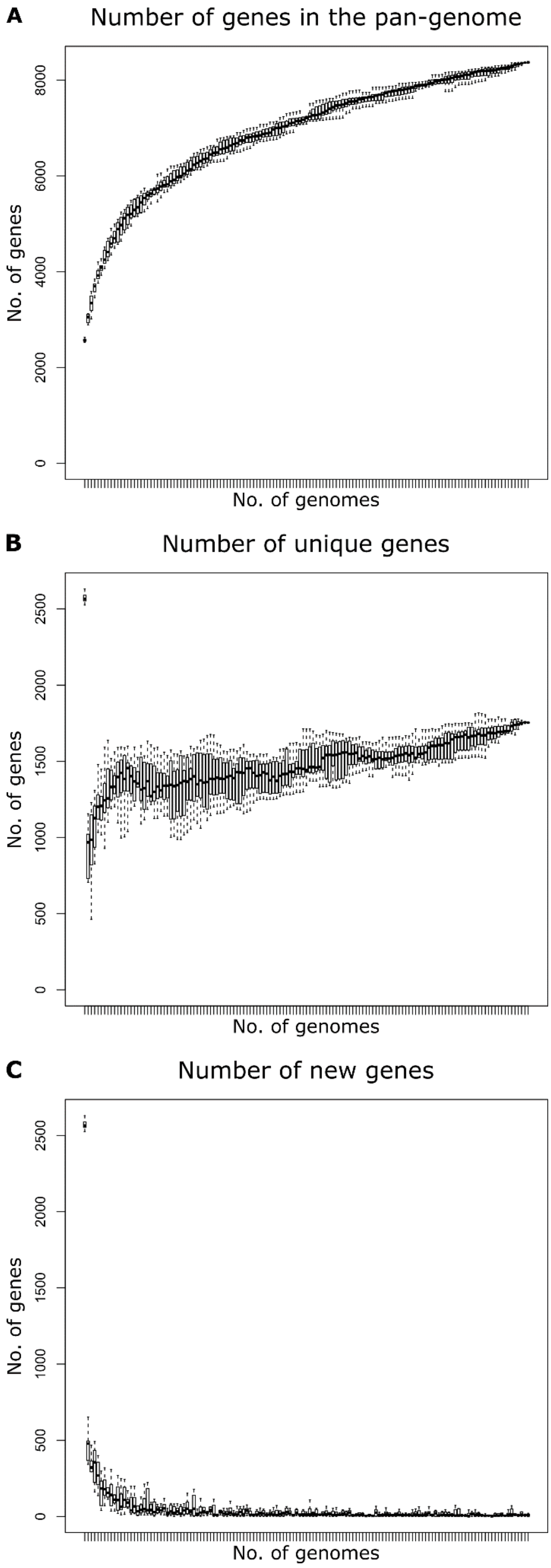


**Fig S1.** Pangenome analysis statistics. The Roary-computed Pangenome of the cohort consists of 8,373 genes (A), displaying a relatively high variability with a large proportion of unique genes (1,754) (B). However, the number of newly added genes per genome quickly drops to very small number (C).

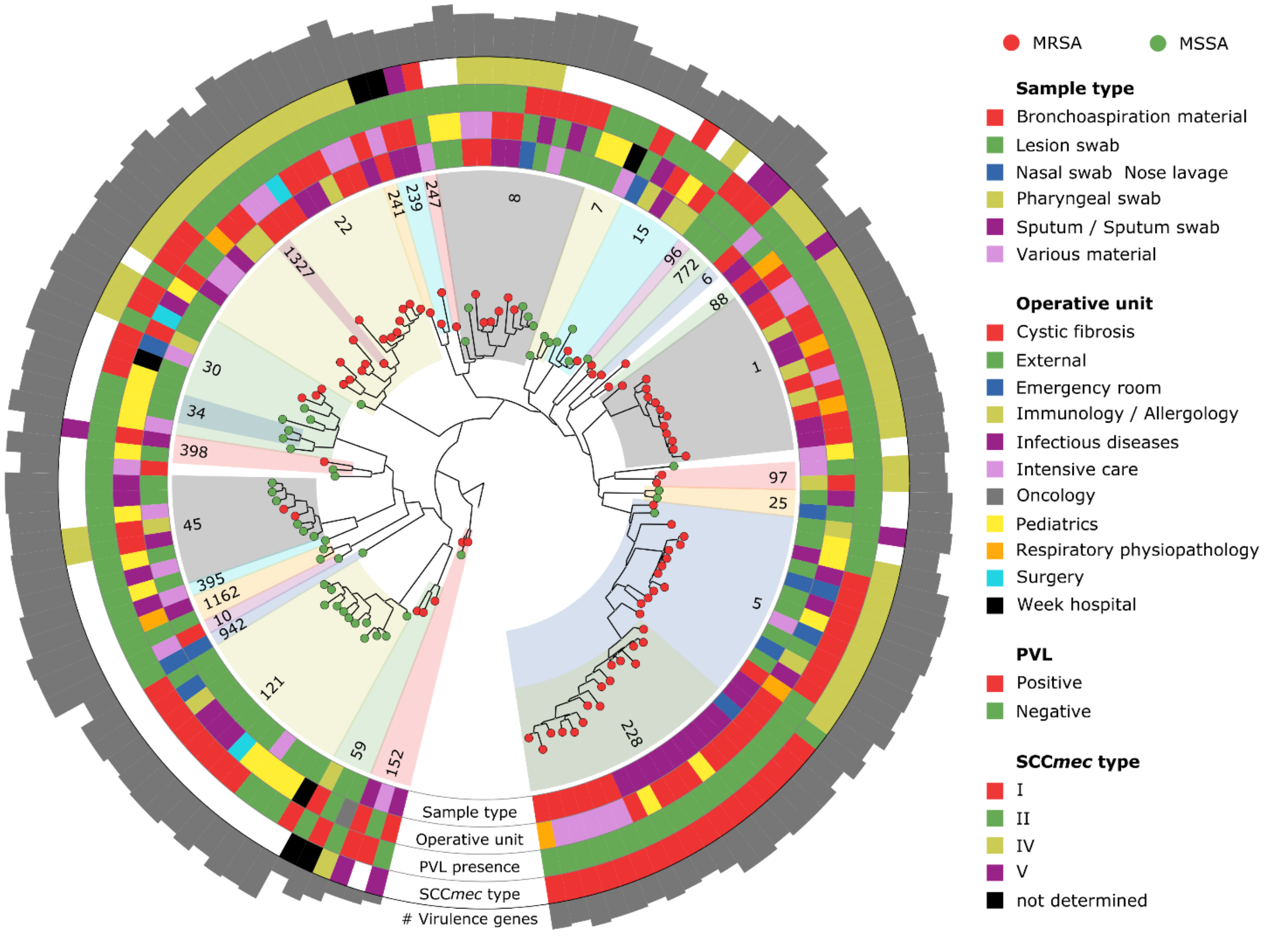


**Fig S2.** Phylogenetic model based on gene presence/absence (1,464 core and 6,909 accessory genes in total) in the 135 single-patient *S. aureus* isolates. STs are distinguished by means of numbers and background colours in the inner ring. Sample type, operative unit, PVL presence, and SCC*mec* type are colour-coded in the following rings. On the outermost ring, the number of virulence genes is reported as bar plot (total considered = 79).
